# Characterization of Equine Chronic Tendon Lesions in Low- and High-Field Magnetic Resonance Imaging

**DOI:** 10.3390/vetsci9060297

**Published:** 2022-06-15

**Authors:** Carla Ulrike Doll, Kerstin von Pueckler, Julia Offhaus, Dagmar Berner, Janina Burk

**Affiliations:** 1 Equine Clinic (Surgery, Orthopedics), Faculty of Veterinary Medicine, Justus Liebig University Giessen, Frankfurter Straße 108, 35392 Giessen, Germany; carla.u.doll@vetmed.uni-giessen.de; 2 Small Animal Clinic (Surgery, Radiology), Faculty of Veterinary Medicine, Justus Liebig University Giessen, Frankfurter Straße 114, 35392 Giessen, Germany; kerstin.h.pueckler@vetmed.uni-giessen.de; 3 Department for Horses, Faculty of Veterinary Medicine, University of Leipzig, An den Tierkliniken 21a, 04103 Leipzig, Germany; julia.offhaus@gmail.com; 4 Equine Referral Hospital, Department of Clinical Science and Services, Royal Veterinary College, Hawkshead Lane, Hatfield AL9 7TA, UK; dberner@rvc.ac.uk

**Keywords:** chronic tendon disease, magnetic resonance imaging, magic angle effect, low-field MRI, high-field MRI

## Abstract

In equine medicine, experience regarding MRI of chronic tendon lesions is limited, and evidence on the suitability of different sequences in 3 T high-field MRI is scarce. Therefore, macroscopically healthy and altered tendons were examined by histology and in 0.27 T low- and 3 T high-field MRI, focusing on T1-weighted (T1w) sequences to visualize chronic lesions. In high-field MRI, tendons were positioned parallel (horizontal) and perpendicular (vertical) to the magnetic field, acknowledging the possible impact of the magic angle effect. The images were evaluated qualitatively and signal intensities were measured for quantitative analysis. Qualitative evaluation was consistent with the quantitative results, yet there were differences in lesion detection between the sequences. The low-field T1w GRE sequence and high-field T1w FLASH sequence with vertically positioned tendons displayed all tendon lesions. However, the horizontally scanned high-field T1w SE sequence failed to detect chronic tendon lesions. The agreement regarding tendon signal intensities was higher between high-field sequences scanned in the same orientation (horizontal or vertical) than between the same types of sequence (SE or FLASH), demonstrating the impact of tendon positioning. Vertical scanning was superior for diagnosis of the tendon lesions, suggesting that the magic angle effect plays a major role in detecting chronic tendon disease.

## 1. Introduction

Tendon disease is one of the most common orthopaedic conditions in sports horses and often adopts a chronic character that predisposes the patients to career-ending re-injuries [1,2]. The superficial digital flexor tendon is most frequently affected, particularly in racehorses [3,4].

To diagnose tendon injuries, ultrasonography is commonly used, but magnetic resonance imaging (MRI) is becoming increasingly important. This trend was fostered by the development of an equine-dedicated low-field MRI system, which has been established around the world over the last twenty years [5]. As this system allows MRI examinations in standing, sedated horses, the risk of a general anaesthesia can be avoided. Compared with ultrasonography, MRI examinations are considered as more accurate in evaluating the extent of tendon damage, whereas tendon lesion size is underestimated in ultrasonographic examinations [6,7]. This implies that MRI is advantageous for long-term follow-up and diagnosis of older tendon lesions and chronic disease [6].

MRI systems with a higher field strength lead to an increased signal-to-noise ratio, offering an even more detailed insight in soft tissues. In human medicine, high-field MRI is gold standard for the diagnosis of tendon pathology [8,9,10]. For high-field examinations in horses, so far mostly 1.5 T systems were used, but settings differed strongly between studies [11,12,13,14]. Studies investigating MRI findings compared with histological appearance showed good agreement with low-field and high-field examinations alike [15,16], but only very few studies investigated the differences between both systems [17].

Independent of the system used, healthy tendons appear hypointense in T1-weighted (w) gradient recalled echo (GRE), T2w and short τ inversion recovery (STIR) spin-echo (SE) sequences, whereas acute lesions are hyperintense in all the sequences mentioned. During the healing process, the signal intensity (SI) of the lesion decreases, especially in T2w and STIR SE sequences over time [11,18]. Consequently, chronic tendon lesions are typically characterized as hyperintense regions in T1w GRE sequences but have a regular or only slightly increased SI in T2w and STIR SE sequences.

Regarding the comparison of low- and high-field MRI systems, different tendon orientation to the magnetic field direction may also matter. Due to the system and the recumbency of the horse in high-field MRI, metacarpal tendon alignment is parallel to the magnetic field direction. In contrast to this, metacarpal tendon alignment is perpendicular to the magnetic field direction in the low-field system for standing horses. This might be relevant because tendons and ligaments share a peculiarity in MRI, as artefacts occur if they are positioned in an angle approximating 55° to the magnetic field direction. This so-called magic angle effect leads to an increased SI in healthy tendons and can cause misinterpretations regarding the presence of lesions [13,19]. The occurrence of magic angle artefacts depends on the sequence chosen, and it is especially provoked at short echo times (TE) [20,21,22]. Therefore, this effect may play a role in the T1w sequences displaying chronic tendon lesions as hyperintense areas.

The aim of this study was to investigate the appearance of chronic tendon lesions in different T1w sequences used in high-field MRI, compared with a T1w GRE sequence in low-field MRI that had already been characterized for equine tendon MRI [23,24]. We hypothesized that not only the higher field strength, but also the position of the tendon fibres to the magnetic field has an influence on the appearance of chronic tendon lesions in MRI.

## 2. Materials and Methods

### 2.1. Tendon Recovery and Selection for MRI

Fifty-six distal forelimbs from adult warmblood horses, disarticulated at the middle carpal joint, were obtained from an abattoir. Superficial digital flexor tendons were dissected, macroscopically evaluated and stored at −20 °C until further examination. Nine tendons with remarkable thickening and eight macroscopically normal tendons were thawed, and ultrasound examinations were performed in order to determine the region to be scanned by MRI.

### 2.2. Magnetic Resonance Imaging

The tendons were fixed in a Plexiglas device to keep them in a straight position. The Plexiglas device was put in a box filled with phosphate buffered saline (PBS). Low-field examinations with the equine-dedicated system (0.27 T MRI unit; Hallmarq Veterinary Imaging, Guildford, Surrey, UK) were performed in a vertical position, corresponding to the position of standing horses. Sequences already established for the investigation of tendon lesions were used (Table 1). A total of 5 healthy and 7 tendons with disease were selected to be additionally examined in a 3 T high-field MRI (Magnetom Verio; Siemens Healthcare GmbH, Erlangen, Germany). Tendons were scanned in a horizontal position as usual for the high-field system (parallel to the magnetic field), using different sequences (Table 2). The T1w SE and fast low-angle shot (FLASH) examinations were additionally performed in a vertical position (perpendicular to the magnetic field), corresponding to the position in low-field MRI.

### 2.3. Histology

For histological verification, one cm long tendon samples, representing either the centre of a lesion or healthy regions, were fixed in 4% paraformaldehyde. To exclude that the tendon lesions investigated were acute, only lesion regions with low signal intensity in the low-field T2 sequence (standardised tendon SI < 2) were chosen. Furthermore, it is of note that after MRI and before fixation, whole tendons had been decellularized as parts of them were subjected to a further study [25]. Four µm thick, longitudinal sections were prepared and subjected to Masson’s trichrome staining to distinguish healthy tendon from the altered tendon matrix within lesion areas [26]. Three images of each section were obtained with a 10× objective, and the percentage of the green-stained area, representative of altered tendon matrix, was determined using Fiji/ImageJ (National Institutes of Health, Bethesda, MD, USA) software.

### 2.4. Qualitative and Quantitative MR Image Analysis

For qualitative evaluation, an equine specialised radiologist scored each image in a blinded manner for the presence or absence of tendon lesions. For this scoring, primary consideration was given to subjectively detectable hyperintense regions within the tendon, as the shape and contour of the tendon could also have preparation-related irregularities, and only images with distinct findings were assigned to the lesion group. For quantitative analysis, the SI of the tendon cross-sectional area was measured in all images using the free-hand tool in Synedra software (Synedra Information Technologies GmbH, Innsbruck, Austria). In images where a lesion was present, the SI of the lesion was measured additionally. In all images, the SI of the background was measured using three 50 mm^2^ circular regions of interest (ROIs) placed lateral, medial and palmar to the PBS-filled box. To standardize the tendon and lesion SIs, the quotient of tendon or lesion SI and the mean SI of the three background ROIs was calculated for each image.

To validate the MRI findings based on the histology results as gold standard, for each histological sample (n = 12), three corresponding MRI images per sequence were analysed. The MRI score for these regions was considered as affected with a lesion if this was true for at least one of the three corresponding MRI images. For SI analyses, the lesion SI was used when a lesion was visible, whereas the tendon SI was used otherwise, and the mean standardised SI from the three corresponding images was calculated.

For all other analyses regarding the MRI findings, the images from the different MRI examinations and sequences were matched, so that the same number of images (n = 195) covering the same anatomic region were available for each sequence, enabling a pairwise analysis. For SI analyses, the standardised tendon SIs were used, irrespective of the presence of a lesion.

### 2.5. Statistical Analysis

Statistical analysis was performed using IBM SPSS Statistics 28. Comparing histological and MRI findings, the 12 histological samples were divided in two groups based on the qualitative score in MRI and the difference in the percentage of green staining between these groups was analysed using the Mann–Whitney U test. Additionally, the Spearman’s rank correlations were tested for the percentages of green staining in histology and the lesion or tendon SIs in MRI. Using the 195 matched images per sequence, the agreements in qualitative lesion detection between the T1w GRE low-field sequence and the different T1w high-field sequences were analysed based on Cohens kappa statistics [27]. To validate the qualitative score, the MRI images were divided in two groups based on their qualitative score and tendon SIs were compared between these groups using the Mann–Whitney U test. For analysing the agreement and differences in tendon SIs between low- and high-field sequences, Spearman’s rank correlations and Wilcoxon signed-rank tests were computed. Additionally, the backtransformed logarithmised limits of agreement, reflecting the mean ratio of SIs between sequences, were calculated [28,29]. *p*-values < 0.05 were considered significant.

## 3. Results

### 3.1. Agreement of MRI Findings in T1w Low-Field and High-Field Sequences with Histology

**Figure 1:**
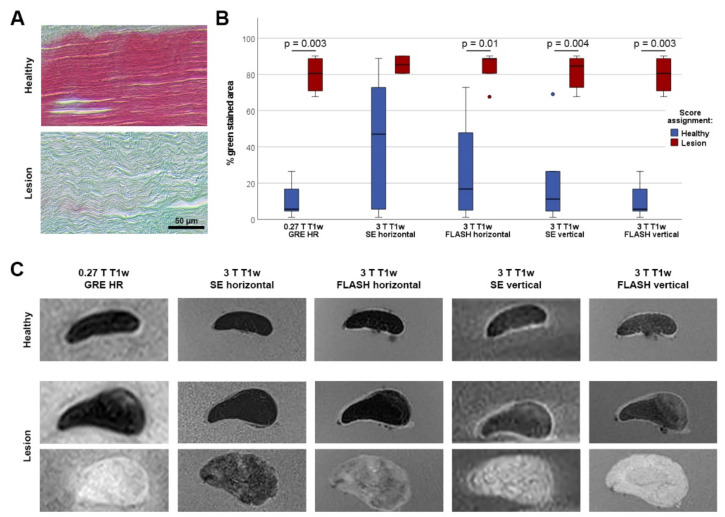
Lesion appearance in histology and MRI. (**A**) Masson’s trichrome staining was performed as gold standard to confirm or to exclude tendon disease. Healthy tendon matrix is stained mainly red, whereas altered tendon matrix is stained green. (**B**) The boxplots display the percentage of green staining in Masson´s trichrome staining, with the groups defined by the score assignment within each sequence. A clear separation of data was obtained using the score assignment from the low-field (0.27 T) T1w GRE HR and the vertically scanned high-field (3 T) FLASH sequences, demonstrating the reliability of lesion detection in these sequences. In case of significant differences between these groups, the *p*-values are indicated. Data were obtained from n = 12 tendon regions. (**C**) The exemplary MRI images show tendon regions that were confirmed to be healthy (upper row) or to be affected with a lesion (middle and lower row) by histology. Correspondingly, the tendon displayed in the upper row shows low signal and was scored as healthy in all T1w sequences, whereas the tendon in the lower row shows high signal and was scored as affected with a lesion in all T1w sequences. However, the tendon in the middle row represents an example of a tendon with a lesion that was not assigned to the lesion group by blinded scoring in the 3 T T1w SE sequence scanned in horizontal orientation, due a lack of distinct signal increase.

Histology revealed that in T1w GRE low-field MRI, all tendon lesions and healthy regions had been correctly identified by blinded qualitative scoring. Tendon regions scored as healthy displayed only low percentages of green-stained area in histology, whereas tendon regions scored as affected with a lesion showed high percentages of green staining (*p* = 0.003) (Figure 1).

Histology data also showed that the reliability of lesion detection in high-field MRI strongly depended on the sequence used. Only the qualitative scoring based on the FLASH sequence in vertical position was as reliable as the scoring based on low-field MRI, with the same difference in histology data (*p* = 0.003). In the horizontally scanned FLASH sequence, as well as in the vertically scanned SE sequence, five and six out of the seven examined lesions had been identified, and the differences in histological green staining were still significant between the two scoring groups (*p* < 0.01) (Figure 1). Scoring based on the horizontally scanned SE sequence yielded the most misleading results, as only two out of seven lesions had been identified by blinded image assessment. These lesions were the only lesions within the histologically examined regions that were also visible in the low-field STIR sequence, suggesting that they still had a higher water content despite their overall low T2 signal intensity.

Quantitative MRI data, namely the lesion or tendon SIs, from all T1w sequences still correlated with the percentage of green staining in histology. The strength of the correlation was similar for the low-field T1w sequence (r = 0.692 and *p* = 0.013) and the high-field T1w FLASH sequences (r = 0.699 and *p* = 0.011 in horizontal position; r = 0.664 and *p* = 0.018 in vertical position), but lower for the high-field SE sequences (r = 0.566 and *p* = 0.055 in horizontal position, r = 0.615 and *p* = 0.033 in vertical position).

### 3.2. Agreement of Qualitative Scoring between T1w Sequences and Measured Signal Intensities

In the low-field T1w GRE sequence, 114 out of the 195 matched images (58.5%) were scored as affected with a lesion. In the vertically scanned high-field T1w FLASH sequence, 134 (68.7%) of all matched images were scored as affected with a lesion, and 87 (44.6%) in the vertically scanned T1w SE sequence. In line with the results described above, the agreement with the T1w GRE low-field sequence regarding qualitative lesion detection was best for the vertical FLASH sequence (89.7% agreement; κ = 0.78; 95% CI: 0.69 to 0.87; *p* < 0.001) and still substantial for the vertical SE sequence (84.1% agreement; κ = 0.69; 95% CI: 0.60 to 0.79; *p* < 0.001).

In contrast, in the horizontally scanned high-field sequences, only 53 (27.2%) of all matched images were scored as affected with a lesion in the T1w SE and 66 (33.9%) in the T1w FLASH sequence. For these, the agreement with the low-field T1w GRE sequence regarding qualitative lesion detection was only moderate, with 68.7% and κ = 0.42 (95% confidence interval (CI): 0.31 to 0.51; *p* < 0.001) for the T1w SE sequence and 74.4% agreement and κ = 0.51 (95% CI: 0.41 to 0.62; *p* < 0.001) for the T1w FLASH sequence.

**Figure 2:**
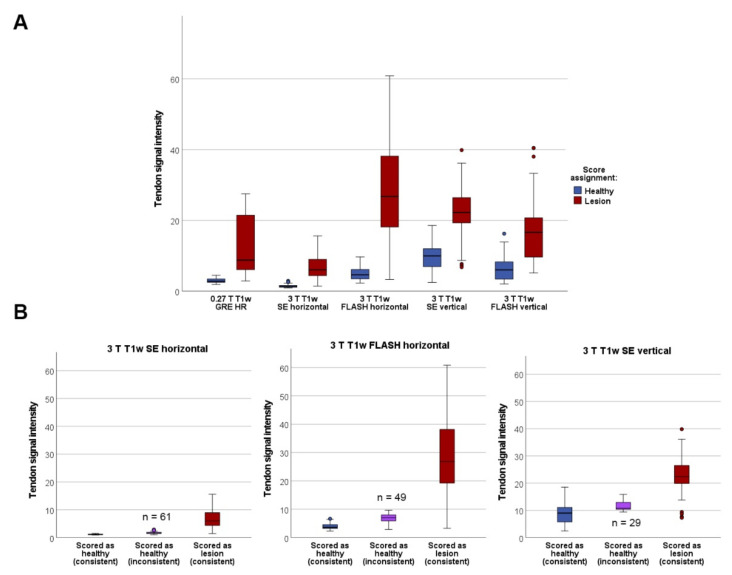
Tendon signal intensities by score assignment. (**A**) Tendon signal intensities in MRI images scored as affected with a lesion were significantly higher than in images scored as healthy, with *p* < 0.001 for all T1w sequences. However, more overlap between groups and more outliers were present in the high-field sequences. (**B**) In the high-field (3 T) T1w SE and FLASH sequences in horizontal position as well as in the SE sequence in vertical position, the score assignment was not consistent with the more reliable low-field (0.27 T) score in the indicated numbers of images. These were scored as healthy in the respective high-field T1w sequences, although a lesion was visible in the low-field T1w GRE sequence. Tendon signal intensity in these inconsistently scored images was as low or nearly as low as in the images that were scored as healthy across all T1w sequences. Data were obtained from n = 195 matched images.

Irrespective of the discrepancies between sequences regarding the identification of the tendon lesions in matched images, the qualitative scoring reflected the differences in the SIs well. In all T1w sequences, the tendon SIs in images scored as affected with a lesion were significantly higher than in images scored as healthy (*p* < 0.001). As illustrated above, in the horizontal SE, horizontal FLASH and vertical SE sequences, there were several images that had been scored as healthy, whereas their more reliable low-field counterparts had shown a lesion. Demonstrating that qualitative judgement was not mistaken, the tendon SIs in these images were equivalent or only slightly higher than in truly healthy tendons (Figure 2).

### 3.3. Agreement of Signal Intensities between Low- and High-Field Sequences

**Figure 3:**
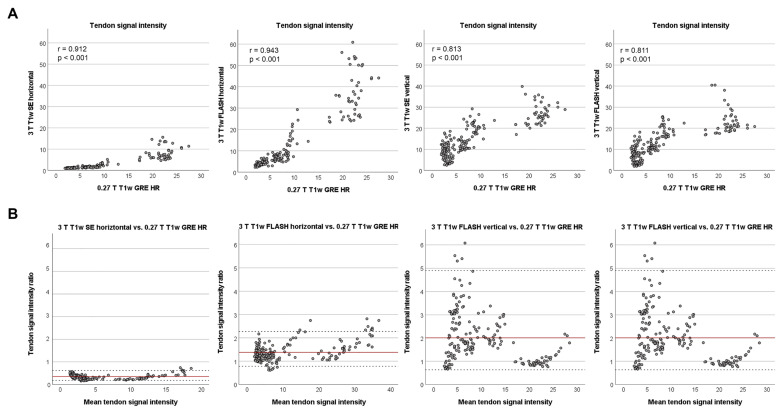
Relationship between tendon signal intensities in T1w high- and low-field MRI. (**A**) The dot plots display the tendon signal intensity in all T1w high-field (3 T) sequences versus tendon signal intensity in the low-field (0.27 T) T1w GRE sequence. All correlations were strong and highly significant. (**B**) The Bland–Altman plots demonstrate that only the high-field SE sequence in horizontal orientation had consistently lower signal intensities than the low-field T1w GRE sequence. The other T1w high-field sequences displayed on average higher signal intensities than the low-field T1w GRE sequence. Data were obtained from n = 195 matched images.

**Figure 4:**
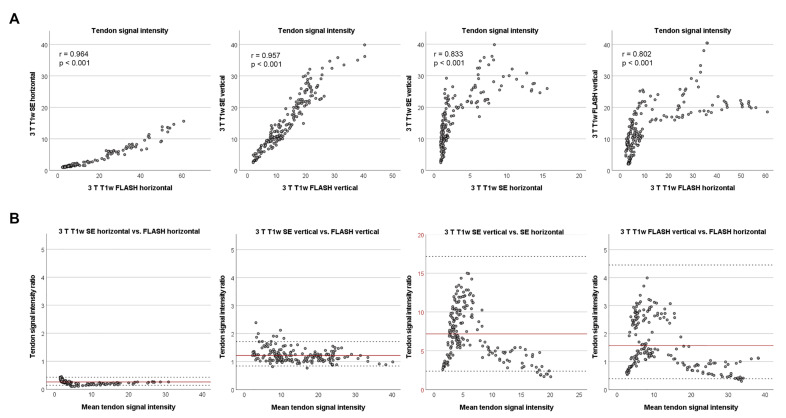
Relationship between tendon signal intensities in the different T1w high-field sequences. (**A**) The dot plots display the tendon signal intensity in the high-field (3 T) SE versus the FLASH sequences in the same orientation, as well as the tendon signal intensities in the SE and FLASH sequences in vertical versus horizontal orientation. Correlations between the different T1w high-field sequences in the same orientation were stronger and the relationship more linear than those between the same type of sequence in different orientations. (**B**) The Bland–Altman plots demonstrate that the high-field SE sequence in horizontal orientation had consistently lower signal intensities than the horizontal FLASH sequence, and that the vertical sequences had a higher signal intensity than their corresponding horizontal sequence (note the different scale in the SE sequence plot). The lower range of variation in the ratio of signal intensities between sequences in the same orientation underlines their better agreement. Data were obtained from n = 195 matched images.

The tendon SIs in all high-field T1w sequences correlated strongly with the low-field T1w sequence (*p* < 0.001) (Figure 3). Regarding the high-field T1w sequences only, interestingly, the correlations between different sequences scanned in the same orientation were stronger than between the same sequence scanned in different orientations (Figure 4). Consequently, the orientation of the tendon to the magnetic field had more impact on the correlation of SIs than the choice of T1w sequence. Furthermore, the dot plots revealed that the relationship between SIs in the same sequence but different orientation was not linear.

Despite the strong correlations between all T1w sequences, the whole tendon SIs differed significantly between them (*p* < 0.001). To determine the extent of difference, the backtransformed logarithmised limits of agreement, representing the ratio of the SIs, were calculated. In the vertically scanned high-field T1w sequences, as well as in the horizontally scanned FLASH sequence, the tendon SIs were, on average, higher than in the low-field sequence. However, the SE sequence in horizontal orientation consistently showed lower tendon SIs than the low-field T1w sequence (Figure 3). 

Regarding the SI ratios of the different high-field T1w sequences to each other, the SIs in the horizontal SE sequence were also lower than in the horizontal FLASH sequence. Furthermore, it was conspicuous that the ratio of the vertical SE sequence to the horizontal SE sequence was much higher than the other ratios, and that the confidence intervals for horizontal vs. vertical sequences were much wider (Figure 4). Accordingly, and in line with the correlation of SIs, the extent of variation of the SI ratio is more strongly influenced by the orientation than by the sequence used. 

Data from T2w and STIR sequences are presented in Supplement S1.

## 4. Discussion

In this study, we investigated the suitability of 3 T high-field T1w SE and FLASH sequences for the detection of chronic tendon lesions, in comparison with a 0.27 T low-field T1 GRE sequence that is already well-established for the examination of equine tendon disease. Since the main difference between both systems, apart from the field strength, is the orientation of the limb in the magnetic field, we performed the high-field examinations with the tendon positioned not only in horizontal, but also in vertical orientation. Our data support the use of GRE sequences in favour of SE sequences and underline the relevance of tendon positioning to achieve an accurate diagnosis of chronic tendon lesions.

Comparing low- and high-field MRI and validating the results based on histology, a similar and high reliability of chronic tendon lesion detection was observed for the T1w GRE low-field sequence and the vertically scanned T1w FLASH high-field sequence, which represents the most analogous high-field GRE sequence investigated in the current study. This may appear surprising considering the lower resolution and signal-to-noise ratio of the low-field MRI and stands in contrast to a previous study, in which visibility of small lesions was better in high-field than in low-field MRI [17]. However, it must be acknowledged that this low-field sequence has already been demonstrated to be suitable for long-term follow-up of tendon lesions [18], and that motion-related artefacts which typically represent a challenge in standing low-field MRI [30] did not play a role in the current study.

Considering the different T1w high-field sequences used, our study supports the use of GRE/FLASH sequences instead of SE sequences for tendon examinations. With respect to diagnosing chronic tendon disease, especially the horizontally scanned T1w SE sequence cannot be recommended, as it failed to display chronic lesions in a distinct manner. This was not only an issue in qualitative scoring, as the scoring results corresponded well with the quantified tendon SI, even when not in line with the actual presence or absence of a lesion. Nevertheless, those lesion areas that showed an increased SI in the low-field STIR sequence also had an increased SI in the horizontally scanned T1w SE sequence, suggesting that more acute lesions are still displayed due to their high water content. The suitability of FLASH sequences for the examination of tendons was already demonstrated for the human Achilles tendon [8,31]. However, high-field SE sequences are also used in 3 T MRI systems for the diagnosis of tendon disease [10,32]. In equine medicine, SE [11,12,13] and FLASH sequences [11,14] have been used equally in 1.5 T MRI systems for the diagnosis of tendon disease. Nevertheless, in accordance with our results, lesions were less conspicuous in SE sequences than in FLASH sequences [11].

Interestingly, the orientation of the tendon to the magnetic field played a more important role than the choice of sequence. To the authors’ knowledge, no studies have focused on this issue yet, as the magic angle effect is normally relevant when tendons or ligaments are positioned oblique, approximating 55°, to the magnetic field. However, we found substantial differences between horizontal (0° to magnetic field) and vertical (90° to magnetic field) positioning of the tendons, along with a strongly improved detection of chronic lesions in the latter. We attribute this to the magic angle effect, not caused by the whole tendon but by newly built collagen fibres. In healthy tendons, the fibres are orientated widely in parallel, therefore, no fibres meet the magic angle orientation in either the horizontal or vertical positions. However, once an injury has occurred, newly built fibres are more randomly orientated [33,34]. Depending on the positioning of the whole tendon, these newly built collagen fibres within the tendon could then be arranged at an angle of about 55° to the magnetic field. When the tendon is then positioned horizontally (0°), a deviation of fibre alignment from the whole tendon direction of 55° would be necessary to achieve the maximum magic angle effect. In contrast, when tendons are positioned vertically (90°), a deviation of fibre alignment from the whole tendon direction of 35° would be sufficient to achieve the maximum magic angle effect, as the 55° angle between fibres and magnetic field would already be met. Presumably, the dependency of MRI findings on the tendon position and magic angle effect is more relevant for scarred, chronic tendon lesions, whereas in acute lesions, inflammation-related tissue properties are responsible for their increased signal [11]. This would explain why the authors of previous studies on equine tendon MRI had experienced less difficulty with lesion detection in T1w SE high-field MRI sequences. This is also supported by a study in which lesions were made visible as less hyperintense regions in the otherwise hyperintense tendon when scanned in a 55° angle to the magnetic field [13]. Probably, in this case, the lesion appeared more hypointense as fewer fibres were orientated at the magic angle. To improve the detection of chronic tendon lesions by high-field MRI, as vertical positioning is not feasible in vivo, it might be considered to position the tendon in question in an oblique way to provoke the magic angle effect in the newly built tendon scar fibres. 

## 5. Conclusions

T1w GRE/FLASH sequences were reliable for detecting chronic tendon lesions, in low- as well as in high-field MRI. However, the T1w SE high-field sequence scanned in the standard horizontal position did not sufficiently capture chronic lesions. Vertical positioning of the tendon strongly improved the reliability of chronic tendon lesion detection, possibly due to randomly orientated fibres susceptible to the magic angle effect.

## Figures and Tables

**Table 1:** MRI sequences used for 0.27 T low-field MRI.

Sequence	Plane	Matrix	FOV ^1^ (mm)	TE(ms)	TR ^2^ (ms)	Slice Thickness (mm)	Gap (mm)
T1w GRE HR ^3^	transverse	512 × 512	169.98	8	78	3.5	0.7
STIR FSE ^4^ HR	transverse	512 × 512	169.98	29	3968	3.5	0.7
T2w FSE HR	transverse	512 × 512	169.98	87	2475	3.5	0.7

^1^ field of view. ^2^ repetition time. ^3^ high resolution. ^4^ fast spin echo.

**Table 2:** MRI sequences used for 3 T high-field MRI.

Sequence	Plane	Matrix	FOV ^1^ (mm)	TE (ms)	TR ^2^ (ms)	Slice Thickness (mm)	Gap (mm)
T1w SE horizontal	transverse	512 × 512	170	12	449	3.5	0.7
T1w 3D trans/SE vertical	transverse	256 × 256	170	8.9	600	0.7	0
Fl2d/FLASH horizontal	transverse	512 × 512	170	4.56	590	3.5	0.7
Fl2d/FLASH vertical	transverse	512 × 512	170	4.56	590	3.5	0.7
STIR SE	transverse	512 × 512	170	51	4100	3.5	0.7
T2w TSE ^3^	transverse	512 × 512	170	118	2475	3.5	0.7

^1^ field of view. ^2^ repetition time. ^3^ turbo spin echo.

## Data Availability

The data presented in this study will be made openly available.

## Supplementary Materials

The following supporting information can be downloaded at: https://www.mdpi.com/article/10.3390/vetsci9060297/s1, Supplement S1: Data from T2w and STIR sequences.
