# Supplementary material for: Characterization of Equine Chronic Tendon Lesions in Low- and High-Field Magnetic Resonance Imaging
Source: Vet Sci. 2022 Jun 15;9(6):297. doi: 10.3390/vetsci9060297 (PMC9229038; doi:10.3390/vetsci9060297)
Supplement: Supplementary file 1 [file vetsci-09-00297-s001.zip › vetsci-1756717-supplementary.pdf]

Supplement S1 to Doll et al., "Characterization of equine chronic tendon lesions in low- and high-field magnetic resonance imaging"

MRI findings in T2w and STIR SE sequences

In the T2w and STIR SE sequences, very few images displayed tendon lesions, confirming the predominantly chronic character of the tendon lesions analysed in this study. All images corresponding to the histologically analysed regions showed a standardized tendon SI < 2 in the low-field T2w SE sequence, as defined as prerequisite for these samples. The low-field STIR SE sequence showed slightly increased signal in two out of twelve histologically analysed regions, which corresponded to the lesions also detected by the horizontally scanned high-field T1w SE sequence. Some further images with higher SI in the T2w and STIR SE sequences were obtained from a tendon which additionally showed acute tendon disease in a region below the histological sections. Due to the resulting scarcity of images scored as affected with a lesion in these sequences, the qualitative scoring results were not statistically compared.

Agreement of T2w and STIR SE tendon signal intensities between low- and high-field MRI

The tendon SIs in T2w and STIR SE sequences correlated between low- and high-field MRI ($p < 0.001$), but the correlation was only moderate for the T2w SE sequences. Furthermore, as observed with the T1w sequences, the tendon SIs in low- and high-field sequences differed significantly from each other ($p < 0.001$). The SIs in the high-field sequences were overall lower than in the corresponding low-field sequences. Data are shown in the figure below.

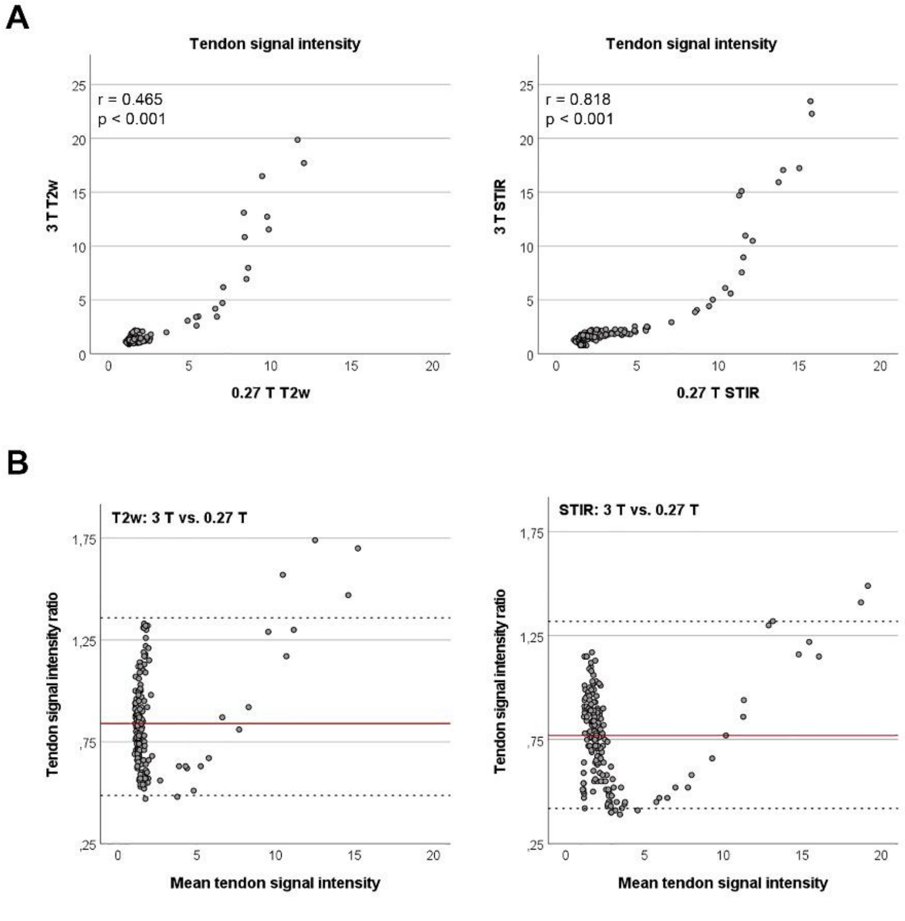

Figure S1: Relationship between tendon signal intensities in T2w and STIR SE high- and low-field MRI. (A) The dot plots display the tendon signal intensity in the high-field (3 T) sequences versus tendon signal intensity in the low-field (0.27 T) sequences. The correlations were significant, but a strong correlation was only found for the STIR sequences. (B) The Bland-Altman-plots demonstrate that the high-field sequences had on average lower signal intensities than the low-field sequences. Data were obtained from n = 195 matched images.
